# A Case of Rectus Sheath Hematoma Caused by Self-Injection of Certolizumab Pegol

**DOI:** 10.7759/cureus.80154

**Published:** 2025-03-06

**Authors:** Atsuhiko Suenaga, Yuka Hashimoto, Akiko Kanaya, Kennichi Fukunari, Yasuhiro Kawahara, Motoaki Miyazono

**Affiliations:** 1 Department of Nephrology, National Hospital Organization Ureshino Medical Center, Ureshino, JPN; 2 Division of Nephrology, Saga University Faculty of Medicine, Saga, JPN; 3 Department of Nephrology, Sasebo Kyosai Hospital, Sasebo, JPN; 4 Department of Radiology, Sasebo Kyosai Hospital, Sasebo, JPN; 5 Department of Nephrology, Saga University, Saga, JPN

**Keywords:** abdominal pain, certolizumab pegol, end-stage kidney disease (eskd), hemodialysis, rectus sheath hematoma

## Abstract

Rectus sheath hematoma (RSH) is a condition caused by hemorrhage into the rectus sheath. A 61-year-old man with end-stage renal failure and rheumatoid arthritis presented to our hospital with abdominal pain. The patient had been receiving biweekly self-injections of certolizumab pegol to the abdominal wall. Computed tomography (CT) demonstrated a large high-density left-sided RSH. Abdominal pain gradually exacerbated; thus, transcatheter arterial embolization (TAE) for the left inferior epigastric artery (IEA) was performed. This is the first reported case of RSH caused by certolizumab pegol. Factors contributing to RSH include low abdominal wall fat, the use of auto-injector-based preparations, and anticoagulant use in dialysis.

## Introduction

Rectus sheath hematoma (RSH) is a relatively uncommon condition. RSH accounts for approximately 2% of patients presenting to the emergency department with acute abdominal pain, and the incidence in patients referred for radiographic examination of abdominal pain is reported to be approximately 1.2-1.5 cases per year [1]. Although no reports have investigated its prevalence, RSH tends to occur more frequently in older women than in men, which is related to increased incidence of chronic disease, increased use of anticoagulation therapy, and repetitive low- and high-intensity trauma [2-4]. RSH is a condition caused by hemorrhage into the rectus sheath. It can be associated with injury of the arteries in the rectus sheath, including the superior epigastric artery, the inferior epigastric artery (IEA), and their branches, or by direct injury to the rectus sheath [5]. There have also been reports of RSH occurring during dialysis, and one of the proposed causes was the use of anticoagulants [6-9]. Certolizumab pegol is a TNF-α inhibitor and is indicated for the treatment of inflammatory autoimmune diseases, such as Crohn’s disease and rheumatoid arthritis. The drug is administered subcutaneously [10]. We herein report the first case of RSH in a hemodialysis patient with an arteriovenous fistula caused by self-injection and administration of certolizumab pegol.

## Case presentation

A 61-year-old man presented to the emergency department with severe abdominal pain five hours after hemodialysis with heparin. The dialysis session that day was completed as normal, and there was no abdominal trauma between the end of dialysis and the patient’s arrival at the hospital. His medical history included end-stage renal failure of unknown etiology for the past 10 years on hemodialysis, rheumatoid arthritis, and short bowel syndrome due to traffic trauma. He was thin due to malabsorption of nutrients caused by short bowel syndrome. He was diagnosed with rheumatoid arthritis 10 years ago and had been treated with analgesics, interleukin-6 inhibitors, interleukin-6 receptor inhibitors, and Janus kinase inhibitors. However, due to side effects, he had been receiving self-injections of the TNF-alpha inhibitor certolizumab pegol to the abdominal wall biweekly after hemodialysis for the past year. The patient experienced challenges in self-administering subcutaneous injections due to rheumatoid arthritis and impaired hand function. Consequently, a healthcare professional was tasked with administering the subcutaneous injections, alternating between the right and left abdominal wall sites. The patient was not taking any anticoagulant or antiplatelet medication except for heparin during hemodialysis. On admission, he was 167 cm tall and weighed 49.5 kg. His body mass index (BMI) was 17.7. His blood pressure was 123/66 mmHg, pulse rate was 89 beats per minute, and body temperature was 36.8°C. Triage tag was green. Physical examination revealed a palpable mass in the left side of the lower abdomen with tenderness. No abnormalities of the heart, lungs, and lower legs were found. There was no blood in the stool or hematemesis. A central venous port was placed in the left anterior thoracic region for central venous nutrition for short bowel syndrome. Blood tests are shown in Table 1. Hemoglobin (Hb) level was measured at 8.2 g/dL. This represents a 1.0 g/dL decrease from the pre-dialysis Hb level of 9.2 g/dL recorded two days prior. However, it is important to note that the actual reduction in Hb concentration may be slightly more pronounced than this observed difference suggests. This is due to the potential hemoconcentration effect resulting from fluid removal during the dialysis process, which could have artificially elevated the post-dialysis Hb measurement. 

**Table 1:** Laboratory findings on admission

Parameter	Patient value	Reference range
White blood cells	4,470/μL	3,300-8,600/μL
Red blood cells	2.98 × 10^6^/μL	3.86-4.92 × 10^6^/μL
Hemoglobin	8.2 g/dL	11.6-14.8 g/dL
Hemoglobin 2 days prior	9.2 g/dL	11.6-14.8 g/dL
Hematocrit	26.60%	35.1-44.4%
Platelet	13.9 × 10^4^/μL	15.8-34.8 × 10^4^/μL
Prothrombin time	77.80%	70-130%
Prothrombin time-international normalized rate	1.14	0.85-1.15
Activated partial thromboplastin time	35.6 seconds	20-40 seconds
Fibrinogen	182 mg/dL	200-400 mg/dL
D-dimer	1.2 μg/mL	<1.0 μg/mL
Antithrombin Ⅲ	46.10%	70-130%
Total protein	4.3 g/dL	6.6-8.1 g/dL
Albumin	2.0 g/dL	4.1-5.1 g/dL
Blood urea nitrogen	9.0 g/dL	8-20 g/dL
Creatinine	3.35 mg/dL	0.46-0.79 mg/dL
Estimated glomerular filtration rate	15.9 mL/min/1.73 m^2^	>60 mL/min/1.73 m^2^
Aspartate aminotransferase	17 IU/L	13-30 IU/L
Alanine aminotransferase	12 IU/L	7-23 IU/L
Lactate dehydrogenase	256 IU/L	124-222 IU/L
Creatine phosphokinase	111 IU/L	41-153 IU/L
Sodium	138 mEq/L	138-145 mEq/L
Potassium	3.7 mEq/L	3.6-4.8 mEq/L
Chloride	108 mEq/L	101-108 mEq/L
C-reactive protein	0.61 mg/dL	0.00-0.14 mg/dL

**Figure 1:**
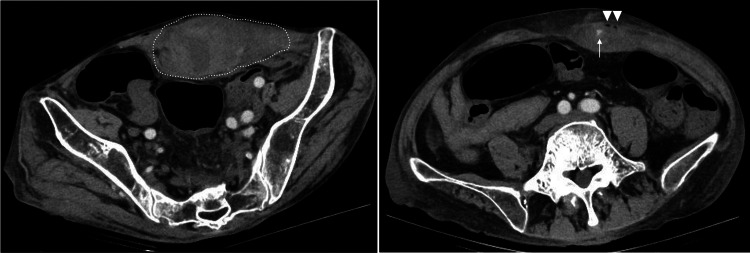
Contrast-enhanced computed tomography (a,b) There was a high-density left-sided RSH of 120 mm × 40 mm (a, dotted line), with small amounts of gas (b, arrow head) and extravasation of contrast media (b, arrow).

**Figure 2:**
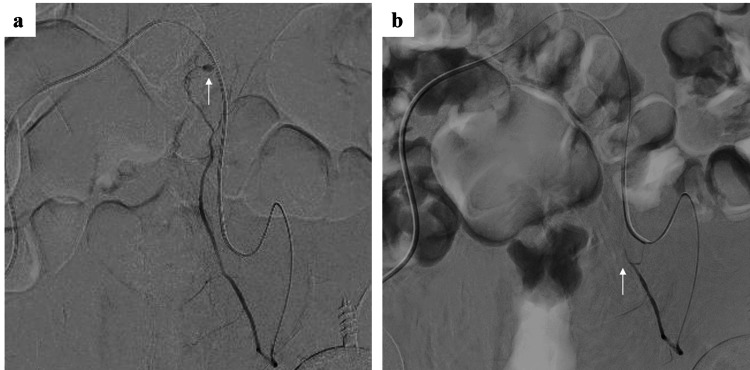
Left inferior epigastric angiography (a) Contrast extravasation was clearly depicted (arrow). Transcatheter arterial embolization (TAE) of the left IEA was performed. (b) After TAE, occlusion of the left IEA with disappearance of contrast extravasation was obtained (arrow).

Abdominal ultrasound showed a hematoma within the left rectus abdominis muscle. Plain computed tomography (CT) taken one hour after the patient’s admission demonstrated a large high-density left-sided RSH of 120 mm × 40 mm with a small amount of gas. On contrast-enhanced CT, extravasation of contrast media was also noted within the RSH (Figure 1). Therefore, we considered that the RSH was caused by vascular injury related to self-injection to the abdominal wall and anticoagulation therapy during hemodialysis. Thirty minutes after the CT, the patient received a transfusion of two units of packed red blood cells because of the possibility of further progression of anemia. The hemodynamics were stable, but abdominal pain gradually exacerbated. Contrast-enhanced CT showed extravasation, and we expected hemodynamic instability in the future. Thus, we considered transcatheter arterial embolization (TAE) necessary for hemostasis. TAE was started one hour after the CT. Angiography demonstrated contrast extravasation from the left IEA (Figure 2a). TAE for the left IEA was performed using gelatin sponge, and contrast extravasation disappeared (Figure 2b). Following the TAE, the patient experienced a significant reduction in abdominal pain on the first post-TAE day. However, Hb levels decreased to 7.6 g/dL. Consequently, two units of red blood cells were transfused. From the second day post-TAE, no further deterioration in anemia was observed. While the hematoma was present, the anticoagulation during hemodialysis was changed to nafamostat mesylate, which has a shorter half-life and fewer bleeding complications. Contrast-enhanced CT on day 6 of TAE showed that the hematoma had shrunk to 65 mm × 31 mm, and there was no extravasation of contrast medium. There were no infectious complications. Clinical symptoms, including pain and palpable masses, gradually improved and fully resolved within 10 days post-TAE. The patient changed the subcutaneous injection site to the thigh area and continued treatment.

## Discussion

RSH, a relatively rare condition, is often a misdiagnosed cause of acute abdominal pain. RSH has been reported to account for approximately 2% of patients presenting to the emergency department with acute abdominal pain [1]. In a study by Klingler et al. in which 1257 patients with acute abdomen were screened and evaluated by ultrasonography, 23 patients (1.8%) were diagnosed with RSH [11]. One study reported that RSH occurred in three of 2,495 patients (approximately 0.12%) receiving subcutaneous anticoagulation with unfractionated heparin [12], and RSH caused by injection into the abdominal wall in this patient population is very rare. As RSH presents with a variety of signs and symptoms, including acute abdomen, abdominal masses, and pain in the abdominal wall, clinicians need to differentiate between other causes of abdominal pain, such as diverticulitis, biliary stones, tumors, and hernia [13]. Several risk factors for RSH have been reported, including abdominal trauma, abdominal surgery, anticoagulation, severe cough, pregnancy, and interestingly, tetanus [13,14]. There have also been reports of RSH occurring during hemodialysis. Their routine use of anticoagulants places them at high risk for developing RSH [6-9]. Hemodialysis patients frequently exhibit pronounced arteriosclerosis and vascular calcification. In this patient, no calcified lesions were visible on CT or fluoroscopic imaging in the left IEA. The relationship between corticosteroids and the development of RSH has been reported, but to our knowledge the relationship between TNFα inhibitors and the development of RSH or coagulation abnormalities has not [9]. Through this observation, it is important to recognize that RSH can be one of the complications of self-injection to the abdominal wall. To our knowledge, this is the first reported case of RSH caused by self-injection of certolizumab pegol to the abdominal wall, although there have been reports of RSH associated with self-injection preparations, such as enoxaparin [15] and insulin [16]. The patient was thin and had little fat in the abdominal wall. The lack of abdominal wall fat was considered one of the factors related to the injury of the left IEA by self-injection to the abdominal wall, and the continuous bleeding without spontaneous hemostasis was associated with the anticoagulant use in dialysis. We also hypothesized that the use of auto-injector-based certolizumab pegol, which prevented adjusting the depth of injection, played a role in arterial injury of the rectus sheath. Immediate CT is needed for the prompt diagnosis of RSH. The most commonly reported abdominal wall hematoma is RSH, although oblique muscle hematoma is also reported in rare cases [17]. Cherry and Mueller reported that of 126 patients treated for RSH at the Mayo Clinic, 114 (86%) were successfully treated with symptomatic therapy and blood transfusion, 10 (7.9%) underwent surgery or endovascular embolization of bleeding vessels, and two (1.6%) died of RSH bleeding [5]. Thus, the majority of patients can be treated conservatively. However, patients with unstable hemodynamics or enlarged hematoma require invasive treatment by TAE or surgical ligation of the bleeding arteries [18]. The patient had an enlarged hematoma and worsening pain, and TAE was deemed necessary. RSH is generally considered a benign condition with a relatively good prognosis. However, in patients undergoing anticoagulation therapy, the hematoma can enlarge and become severe more easily. While the mortality rate for RSH is reported to be 4%, there are reports suggesting that in patients on anticoagulation therapy, the fatality rate can increase up to 25% [19]. The patient had received anticoagulation during hemodialysis before the onset of RSH, and contrast-enhanced CT showed extravasation, so TAE was the treatment of choice. Twelve reports of RSH cases treated with TAE have shown that in all cases the bleeding point was identified and hemostasis was achieved, with no cases of rebleeding or TAE-related. There were no cases of rebleeding or TAE-related complications. The role of surgery for RSH is insufficient to compare with TAE due to the lack of literature. Nevertheless, TAE is an effective and safe procedure in the management of RSH to detect and control the source of bleeding when conservative management fails to stabilize hemodynamics [20]. In this case, too, TAE was selected and successfully treated because it is minimally invasive and allows rapid hemostasis compared to open surgery. One method to prevent RSH, as recommended in the literature and in the medication’s prescribing information, such as insulin administration route, is to inject into areas other than the abdominal wall, such as the shoulders or thighs. This patient began receiving subcutaneous injections in the thigh area.

## Conclusions

In conclusion, we reported a rare case of RSH caused by self-injection of certolizumab pegol to the abdominal wall in a hemodialysis patient. Factors contributing to RSH include low abdominal wall fat, the use of auto-injector-based preparations, and anticoagulant use in dialysis. Self-injection into the abdominal wall, regardless of the medication used, can lead to RSH. Clinicians should be reminded that self-injection to the abdominal wall can cause RSH for prompt diagnosis and to avoid misdiagnosis.
